# New data on long-jawed spiders (Araneae, Tetragnathidae, *Tetragnatha*) from China

**DOI:** 10.3897/zookeys.1269.177630

**Published:** 2026-02-13

**Authors:** Changtao Liu, Zongguang Huang, Haiqiang Yin, Xiang Xu

**Affiliations:** 1 College of Life Sciences, Hunan Normal University, Changsha, Hunan 410081, China College of Life Sciences, Hunan Normal University Changsha China

**Keywords:** Morphology, orb-weaving spiders, taxonomy

## Abstract

Two species of *Tetragnatha* Latreille, 1804 are described, including one new species, *Tetragnatha tricuspidata***sp. nov**. (♀♂) from Sichuan and Shaanxi Provinces, and one Chinese newly recorded species, *Tetragnatha hirashimai* Okuma, 1987 from Guangxi Zhuang Autonomous Region and Yunnan Province. Detailed descriptions, photographs, and a distribution map of both species are provided.

## Introduction

The genus *Tetragnatha* Latreille, 1804 was established based on its type species, originally described as *Aranea extensa* Linnaeus, 1758, from Sweden. With a long history of research and a cosmopolitan distribution, *Tetragnatha* is the most species-rich genus in the family Tetragnathidae Menge, 1866, comprising 318 known species or subspecies worldwide (World Spider Catalog 2025). Members of this genus are characterized by exceptionally strong chelicerae and elongate maxillae. Cheliceral morphology is especially important for species identification in *Tetragnatha* because members of the subfamily Tetragnathinae Menge, 1866, which includes *Tetragnatha* and six other genera, have evolved haplogyne genitalia, resulting in the generally simplified female genital structures (Álvarez-Padilla and Hormiga 2011). *Tetragnatha* spiders typically inhabit vegetation near water bodies, such as ponds, streams, and karst springs where they construct horizontal or vertical orb-webs with an open hub, and interestingly, they often dismantle and ingest their webs daily (Dippenaar-Schoeman et al. 2023).

Since *Tetragnatha* spiders are mostly large-bodied and extensively studied, the discovery rate of new species has declined; only 41 valid species have been described worldwide since 2000 (World Spider Catalog 2025). Furthermore, the insufficiently detailed early species descriptions and the lack of comprehensive revision have complicated species identification within the genus.

During the examination of specimens collected over several years by the research team at Hunan Normal University, we identified one new *Tetragnatha* species that is described here. Additionally, we report *T. hirashimai* Okuma, 1987 from China for the first time and redescribe it. With their addition, the number of *Tetragnatha* species known from China increases from 49 to 51.

## Materials and methods

Specimens were examined using an Olympus SZX16 stereomicroscope and an Olympus BX53 compound microscope. Photographs were taken with a Canon PowerShot G12 digital camera mounted on an Olympus BX53 compound microscope, and final multifocal images were generated using HELICON FOCUS 6.0 (https://www.heliconsoft.com). Male palps and female genitalia were dissected for examination and illustration. Female genitalia were digested with pancreatin prior to examination (Álvarez-Padilla and Hormiga 2008). All measurements were obtained using a Leica M205C stereomicroscope. Eye diameters were taken at the widest point. Leg measurements are given as total length of left leg as: femur, patella, tibia, metatarsus, tarsus. Leg segments were measured on their dorsal sides. All measurements are in millimeters (mm). All specimens examined in this study are deposited in the College of Life Sciences, Hunan Normal University (**HNNU**), China.

Terminology used in this paper partially follows Castanheira et al. (2019).

Abbreviations: **a**: male dorsal apophysis; **ALE**: anterior lateral eyes; **AME**: anterior median eyes; **AXl**: auxiliary guide tooth of the lower row; **AXu**: auxiliary guide tooth of the upper row; **AME−ALE**: distance between AME and ALE; **AME−AME**: distance between AME; **C**: conductor; **CB**: cheliceral bulge; **CS**: central membranous sac; **Cy**: cymbium; **E**: embolus; **Gl**: guide tooth of the lower row; **Gu**: guide tooth of the upper row; **K**: knob; **L**: translucent lobe; **Ln**: the nth teeth of the lower row from the distal end (for example, the second teeth is labelled L2), but the first one named Gl; **MC**: median cusp of the fang; **MOA**: median ocular area; **N**: notch; **P**: paracymbium; **PLE**: posterior lateral eyes; **PME**: posterior median eyes; **PME−PLE**: distance between PME and PLE; **PME−PME**: distance between PME; **Sp**: spermatheca; **Un**: the nth teeth of the upper row from the distal end (for example, the second teeth is labelled U2), but the first one named Gu.

## Taxonomy

### Family Tetragnathidae Menge, 1866

**Subfamily Tetragnathinae Menge, 1866**

#### Genus Tetragnatha

Taxon classificationAnimaliaAraneaeTetragnathidae

Latreille, 1804

B7E4AACB-9B59-5EC4-B26A-1A13D3764D56

##### Type species.

*Tetragnatha extensa* (Linnaeus, 1758) from Sweden.

##### Diagnosis.

**Figure 1.**
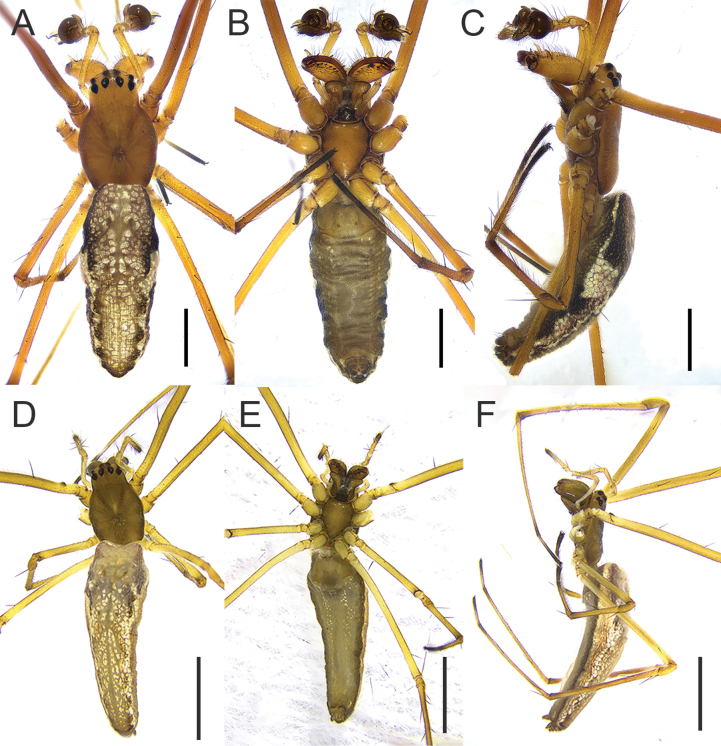
*Tetragnatha tricuspidata* sp. nov., habitus (HNU1092, HNU1111). **A**. Male, dorsal view; **B**. Ditto, ventral view; **C**. Ditto, lateral view; **D**. Female, dorsal view; **E**. Ditto, ventral view; **F**. Ditto, lateral view. Scale bars: 1 mm (**A–C**); 2 mm (**D–F**).

**Figure 2.**
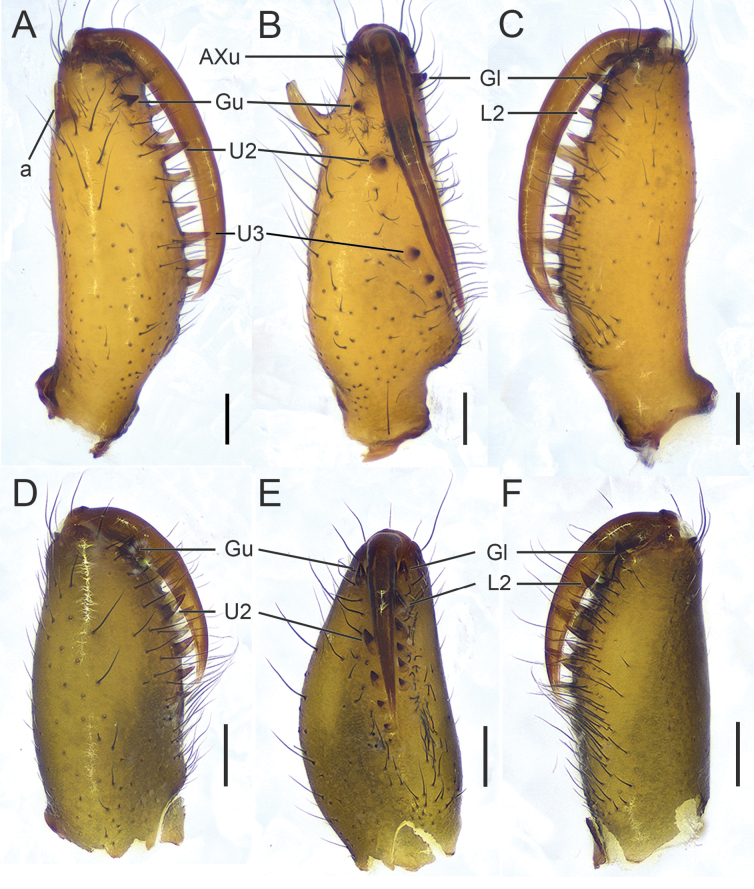
*Tetragnatha tricuspidata* sp. nov., chelicerae (HNU1092, HNU1111). **A**. Male, upper view; **B**. Ditto, inner view; **C**. Ditto, lower view; **D**. Female, upper view; **E**. Ditto, inner view; **F**. Ditto, lower view. Abbreviations: a = male dorsal apophysis, AXu = auxiliary guide tooth of the upper row, Gl = guide tooth of the lower row, Gu = guide tooth of the upper row, L2 = the second teeth of the lower row from the distal end (the first teeth named Gl), U2 = the second teeth of the upper row from the distal end (the first teeth named Gu), U3 = the third teeth of the upper row from the distal end (the first teeth named Gu). Scale bars: 0.2 mm.

This genus resembles *Pachygnatha* Sundevall, 1823 in having elongate chelicerae (Fig. 2) and the absence of a sclerotized epigynum (Figs 1E, 4E, 5G), but it can be distinguished by the following characters: (1) maxillae elongate with widened distal ends in *Tetragnatha*, vs maxillae of normal proportions, not elongate, in *Pachygnatha* (cf. Fig. 1B, E with fig. 1B in Huang et al. 2024); (2) abdomen narrow, elongate, and nearly cylindrical in *Tetragnatha*, vs abdomen oval or spherical in *Pachygnatha* (cf. Fig. 1A with fig. 1A in Huang et al. 2024); (3) lateral eyes lacking tapeta in *Tetragnatha*, vs tapeta present in lateral eyes of *Pachygnatha* (cf. Fig. 1C with fig. 1C in Huang et al. 2024).

#### Tetragnatha tricuspidata sp. nov.

Taxon classificationAnimaliaAraneaeTetragnathidae

0BFC8358-E208-55EA-8DEB-F21F4C746789

https://zoobank.org/2CFFBA0B-6F96-4456-9FAB-58ECBB61EBA2

**Figure 3.**
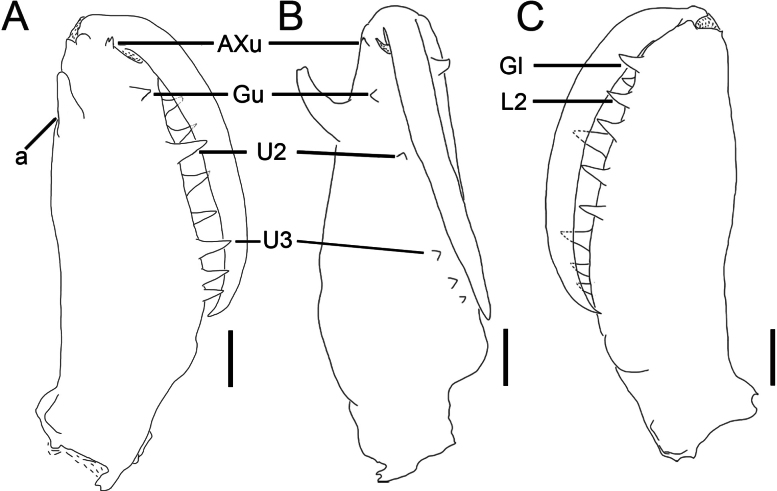
*Tetragnatha tricuspidata* sp. nov., chelicerae, male (HNU1092). **A**. Left chelicera, upper view; **B**. Ditto, inner view; **C**. Ditto, lower view. Abbreviations: a = male dorsal apophysis, AXu = auxiliary guide tooth of the upper row, Gl = guide tooth of the lower row, Gu = guide tooth of the upper row, L2 = the second teeth of the lower row from the distal end (the first teeth named Gl), U2 = the second teeth of the upper row from the distal end (the first teeth named Gu), U3 = the third teeth of the upper row from the distal end (the first teeth named Gu). Scale bars: 0.2 mm.

**Figure 4.**
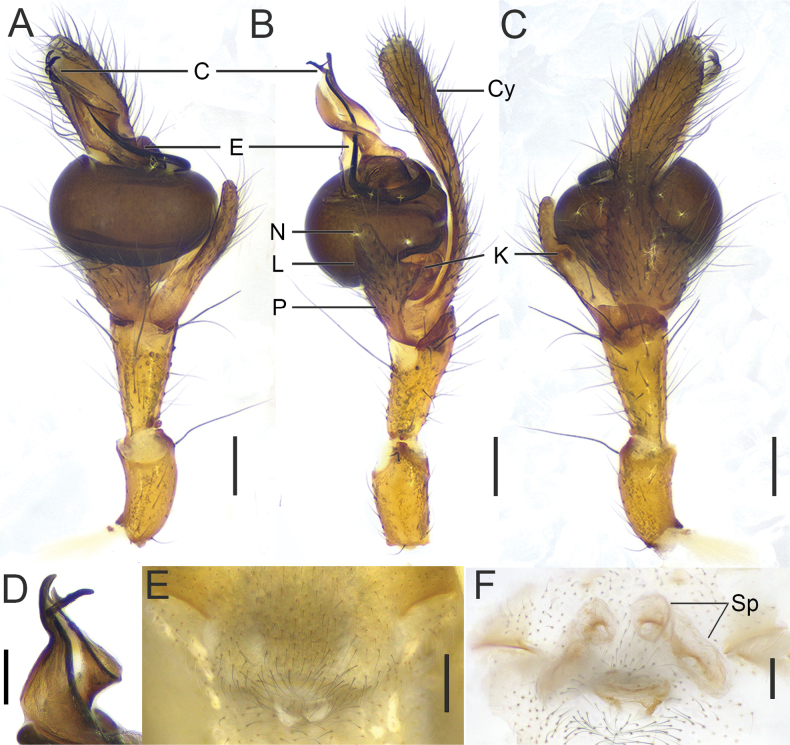
*Tetragnatha tricuspidata* sp. nov., genitalia (HNU1092, HNU1111). **A**. Pedipalp, ventral view; **B**. Ditto, retrolateral view; **C**. Ditto, dorsal view; **D**. Conductor detail, ventroprolateral view; **E**. Genital fold, ventral view; **F**. Vulva, dorsal view. Abbreviations: C = conductor, Cy = cymbium, E = embolus, K = knob, L = translucent lobe, N = notch, P = paracymbium, Sp = spermatheca. Scale bars: 0.2 mm.

Figs 1, 2, 3, 4, 7 Chinese name: 三尖肖蛸

##### Type material.

***Holotype***: China • ♂; Shaanxi Province, Baoji City, Feng County, Ma Village; 33°51'39"N, 106°31'48"E; 1672 m a.s.l.; 6 Jun. 2022; Ailan He, Jinxin Liu, Zongguang Huang, Yun Liang, Yu Hui, Yingli Wen, Yang Liu leg.; HNU1092. ***Paratypes***. China • 1 ♀; Shaanxi Province, Baoji City, Feng County, Tongtianhe River National Forest Park; 32°41'13"N, 106°47'44"E; 2046 m a.s.l.; 5 Jun. 2022; same collectors as holotype; HNU1111 • 1 ♀; Sichuan Province, Bazhong City, Nanjiang County, Guangwushan–Nuoshuihe UNESCO Global Geopark, Micangshan Mountain; 32°39'51"N, 106°55'54"E; 1406 m a.s.l.; 2 Jun. 2022; same collectors as holotype; HNU1113.

##### Diagnosis.

Both male and female of the new species resemble those of *T. bifurcata* Li & Liu, 2022. Males have a similar conductor with two folds and a bifurcated distal end, with the fang slightly curved toward the lower view, and females of the two species are similar in the shape of genital fold and in having short and laterally bulging chelicerae (with the same number of teeth), but the two species differ in the following characters: (1) male dorsal apophysis (a) located near the dorsal surface of the chelicera, away from the promarginal tooth in new species, vs located midway between the promarginal tooth and the dorsal surface of the chelicera in *T. bifurcata* (cf. Fig. 2A with fig. 1D in Li et al. 2022); (2) distal end of conductor with three tips in new species, vs two tips in *T. bifurcata* (cf. Fig. 4D with fig. 1L in Li et al. 2022); (3) the anterior pair of spermathecae are spherical and the posterior pair of spermthecae are oblong in new species, vs both the anterior and posterior pair of spermathecae are spherical in *T. bifurcata* (cf. Fig. 4F with fig. 2H in Li et al. 2022).

##### Description.

**Male (holotype)**. Total length 5.12. Carapace 2.01 long, 1.28 wide, yellowish brown; cervical groove dark; radial grooves indistinct; fovea inverted V-shaped (Fig. 1A). Bases of anterior and posterior lateral eyes contiguous. Eye diameters and interdistances: AME 0.09, ALE 0.08, PME 0.10, PLE 0.09; AME–AME 0.10, AME–ALE 0.17, PME–PME 0.17, PME–PLE 0.18. MOA 0.27 long, anterior width 0.28, posterior width 0.38. Legs slender, similar in colour to carapace (Fig. 1A–C), trichobothria present on femur. Leg measurements: I 19.19 (5.44, 0.67, 5.81, 5.91, 1.36); II 13.10 (3.89, 0.75, 3.73, 3.76, 0.97); III 5.82 (2.02, 0.49, 1.32, 1.42, 0.57); IV 11.34 (3.71, 0.49, 2.90, 3.37, 0.87). Chelicerae yellow, “a” situated near the dorsal surface of chelicera, with flat end; AXu short and bifurcate, AXl absent; upper row with five teeth, Gu smaller than U2, distance between Gu and U2 shorter than that between U2 and U3; lower row with five teeth, Gl equal in size to L2 (Figs 2A–C, 3). Sternum 1.06 long, 0.84 wide, yellow, shield-shaped (Fig. 1B). Maxillae yellowish brown, longer than wide, with widened distal ends. Labium wider than long. Abdomen 3.10 long, 1.23 wide, elliptical, dorsally with irregular silver guanine spots interspersed with brown markings (Fig. 1A, C).

**Palp** (Fig. 4A–D). Tibia twice as long as wide, distal dorsal margin extended into plate-like protuberance. Paracymbium with a triangular knob, a shallow notch, and a translucent lobe occupying almost 1/3 the width of paracymbium in retrolateral view. Cymbium constricted medially, distal portion curled into semi-enclosed configuration. Tegulum like a sphere missing both ends in ventral view. Conductor distal end divided into two branches, ventral branch with an additional median tip, resulting in three tips (in retrolateral view, the additional median tip appears as a short line between branches; in ventro-prolateral view, its full shape is visible) (Fig. 4B, D). Embolus with broad basal half circling clockwise around the bulb, slender distal half extending toward bases of distal tip of conductor (Fig. 4B).

**Female (paratype)**. Total length 6.47. Carapace 1.96 long, 1.25 wide, yellowish green; cervical groove dark; radial grooves indistinct; fovea triangular (Fig. 1D). Eye diameters and interdistances: AME 0.09, ALE 0.07, PME 0.09, PLE 0.09; AME–AME 0.10, AME–ALE 0.19, PME–PME 0.17, PME–PLE 0.16. MOA 0.30 long, anterior width 0.29, posterior width 0.36. Leg measurements: I 18.19 (5.13, 0.72, 5.59, 5.46, 1.29); II 12.23 (3.58, 0.76, 3.20, 3.56, 1.13); III 5.41 (1.76, 0.47, 1.24, 1.29, 0.65); IV 10.49 (3.52, 0.52, 2.58, 2.99, 0.88). Chelicerae slightly green; upper row with five teeth, U2 largest and separated from Gu widely; lower row with six teeth, L2 largest and separated from Gu slightly (Fig. 2D–F). Sternum 1.06 long, 0.82 wide, yellow-green, shield-shaped. Maxillae yellowish green, longer than wide, with widened distal ends. (Fig. 1E). Abdomen 4.51 long, 1.40 wide. Other somatic characters as in male.

**Female genitalia** (Fig. 4E, F). Genital fold wider than long. Two pairs of spermathecae: anterior pair nearly spherical, posterior pair oblong; anterior pair situated between posterior spermathecae. Central membranous sac (CS) absent.

##### Etymology.

The specific epithet is from the Latin *tricuspidatus* (“three-tipped”), referring to the conductor bearing three distal tips; adjective.

##### Distribution.

Known only from the type locality (Fig. 7).

#### Tetragnatha hirashimai

Taxon classificationAnimaliaAraneaeTetragnathidae

Okuma, 1987

DF4ACFB8-BA37-5DF2-B4FC-A80A171A6B50

Figs 5, 6, 7

Tetragnatha hirashimai Okuma, 1987: figs 25, 26A–K.

##### Material examined.

China • 1 ♂ 1 ♀; Guangxi Zhuang Autonomous Region, Baise Prefecture, Jingxi City, Wuling Forest Park; 24 Jun. 2023; 23°4'29"N, 106°26'39"E; 821 m a.s.l.; Jinxin Liu, Zongguang Huang, Yun Liang, Jinnan Liu, Yecheng Wu leg.; HNU1075, HNU1110 • 1 ♀; Yunnan Province, Xishuangbanna Dai Autonomous Prefecture, Mengla County, Xishuangbanna Tropical Rainforest National Park, Wangtianshu Scenic Area; 27 Jun. 2023; 21°37'27"N, 101°35'12"E; 577 m a.s.l.; Jinxin Liu, Zongguang Huang, Yun Liang, Jinnan Liu, Yecheng Wu leg.; HNU520 • 3 ♀♀; Yunnan Province, Xishuangbanna Dai Autonomous Prefecture, Mengla County, Xishuangbanna Tropical Botanical Garden; 30 Jun. 2023; 21°55'1"N, 101°16'14"E; 577 m a.s.l.; Jinxin Liu, Zongguang Huang, Yun Liang, Jinnan Liu, Yecheng Wu leg.; HNU521–523.

##### Diagnosis.

**Figure 5.**
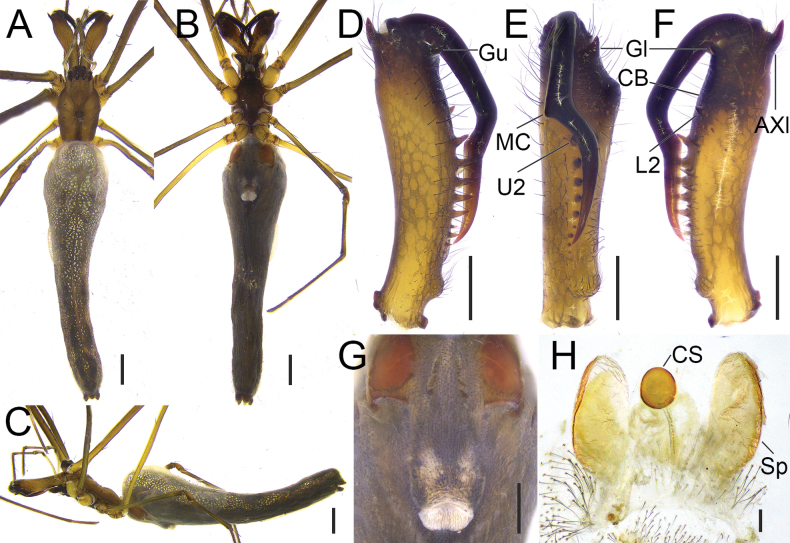
*Tetragnatha hirashimai* Okuma, 1987, female (HNU1075). **A**. Habitus, dorsal view; **B**. Ditto, ventral view; **C**. Ditto, lateral view; **D**. Left chelicera, upper view; **E**. Ditto, inner view; **F**. Ditto, lower view; **G**. Genital fold, ventral view; **H**. Vulva, dorsal view. Abbreviations: AXl = auxiliary guide tooth of the lower row, CB = cheliceral bulge, CS = central membranous sac, Gl = guide tooth of the lower row, Gu = guide tooth of the upper row, L2 = the second teeth of the lower row from the distal end (the first teeth named Gl), MC = median cusp of the fang, Sp = spermatheca, U2 = the second teeth of the upper row from the distal end (the first teeth named Gu). Scale bars: 1 mm (**A–C**); 0.5 mm (**D–G**); 0.1 mm (**H**).

**Figure 6.**
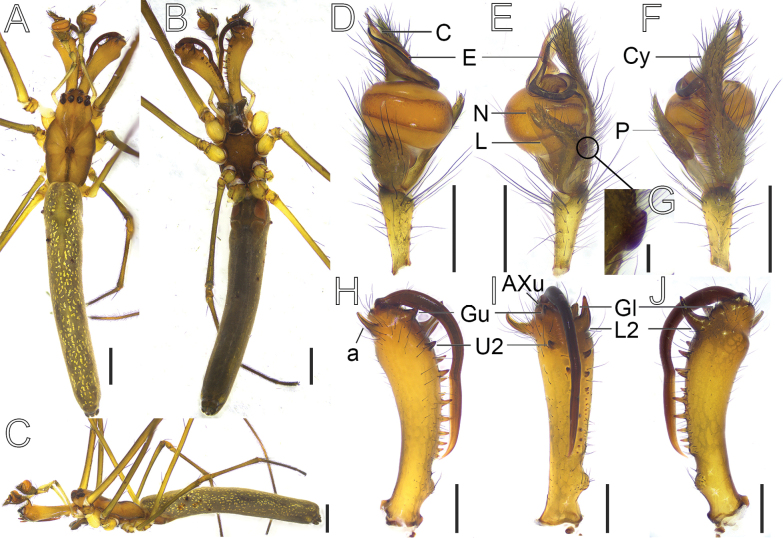
*Tetragnatha hirashimai* Okuma, 1987, male (HNU1110). **A**. Habitus, dorsal view; **B**. Ditto, ventral view; **C**. Ditto, lateral view; **D**. Left pedipalp, ventral view; **E**. Ditto, retrolateral view; **F**. Ditto, dorsal view; **G**. Knob, retrolateral view; **H**. Chelicera, upper view; **I**. Ditto, inner view; **J**. Ditto, lower view. Abbreviations: a = male dorsal apophysis, AXu = auxiliary guide tooth of the upper row, C = conductor, Cy = cymbium, E = embolus, Gl = guide tooth of the lower row, Gu = guide tooth of the upper row, L = translucent lobe, L2 = the second teeth of the lower row from the distal end (the first teeth named Gl), N = notch, P = paracymbium, U2 = the second teeth of the upper row from the distal end (the first teeth named Gu). Scale bars: 1 mm (**A–C**); 0.5 mm (**D–F**); 0.1 mm (**G**); 0.5 mm (**H–J**).

Both males and females of *T. hirashimai* are similar to those of *T. mandibulata* Walckenaer, 1841. Males have a slender abdomen, and similar paracymbium with a translucent lobe occupying half of its width and strongly curved “a” situated near the dorsal surface of chelicerae (Fig. 6E, H), and females have an elongate genital fold, and similar chelicerae with strongly curved fangs, conspicuous cheliceral bulge (CB) and distinct bulky AXl (Fig. 5F), but the two species can be distinguished by: (1) male L2 (the second tooth of the lower row from the distal end) distinctly enlarged compared to the other teeth in this species, vs L2 not enlarged relative to the other teeth in males of *T. mandibulata* (cf. Fig. 6I with fig. 11E in Castanheira et al. 2019); (2) females chelicerae with cusp located at the median position of the fang in this species, vs at the basal position of the fang in females of *T. mandibulata* (cf. Fig. 5E with fig. 12E, F in Castanheira et al. 2019); (3) central membranous sac (CS) spherical, and smaller than spermathecae in species, vs long oval, slightly bigger than spermathecae in females of *T. mandibulata* (cf. Fig. 5H with fig. 12I in Castanheira et al. 2019).

##### Description.

**Female**. Total length 8.92. Carapace 2.45 long, 1.16 wide, yellow-brown; cervical groove distinct; radial grooves indistinct; fovea arc-shaped (Fig. 5A). Anterior and posterior eye rows recurved; lateral eyes close together. Eye diameters and interdistances: AME 0.11, ALE 0.10, PME 0.11, PLE 0.11; AME–AME 0.12, AME–ALE 0.14, PME–PME 0.14, PME–PLE 0.13. MOA 0.33 long, anterior width 0.34, posterior width 0.36. Clypeus 0.07 high. Legs slender, with similar color to the carapace. Leg measurements: I 27.32 (8.61, 0.89, 8.36, 7.53, 2.17), II 16.55 (5.30, 0.71, 4.43, 4.97, 1.14), III 7.61 (2.86, 0.37, 1.67, 2.01, 0.70), IV 16.41 (5.95, 0.58, 3.95, 4.81, 1.12). Chelicerae yellow-brown, bearing a smooth cheliceral bulge (CB) between the upper row and lower row; AXl bulky; upper row with 10 teeth, Gu bulky and separated from U2 widely; lower row with six teeth, Gl slightly thinner than Gu and larger than L2, widely separated from L2; median cusp (MC) situated approximately midway along the length of the fang (Fig. 5D–F). Sternum 1.29 long, 0.91 wide, dark brown. Maxillae dark brown, longer than wide, distal ends widened. Labium dark brown, wider than long. Abdomen 6.46 long, 1.27 wide, dorsally covered with silver guanine spots; anterior one-third slightly swollen, posterior two-thirds slender (Fig. 5A).

**Female genitalia** (Fig. 5G, H): genital fold longer than wide. Spermathecae paired, large and oval, with their outer margin slightly sclerotized. CS spherical, located between spermathecae, connected by a thin stalk.

**Male**. Total length 7.82. Carapace 2.13 long, 1.23 wide, yellowish brown; cervical groove distinct; radial grooves indistinct; fovea arc-shaped (Fig. 6A). Anterior eye row strongly recurved; bases of anterior and posterior lateral eyes contiguous. Eye diameters and interdistances: AME 0.10, ALE 0.10, PME 0.11, PLE 0.10; AME–AME 0.10, AME–ALE 0.14, PME–PME 0.14, PME–PLE 0.15. MOA 0.27 long, anterior width 0.31, posterior width 0.37. Legs slim, with similar color to the carapace (Fig. 6A−C). Leg measurements: I 28.29 (8.93, 0.78, 8.56, 7.73, 2.29), II 16.93 (5.31, 0.75, 4.62, 4.98, 1.27), III 8.10 (2.83, 0.45, 1.86, 2.19, 0.77), IV 16.83 (6.07, 0.61, 3.98, 4.95, 1.22). Chelicerae yellow, the bases of male dorsal apophyses (“a”) perpendicular to chelicerae, with distal ends curved toward the bases of the fangs, AXu small; upper row with nine teeth, Gu bulky, separated from U2; lower row with 11 teeth, Gl small and close to the robust L2 (Fig. 6H−J). Sternum 1.05 long, 0.82 wide, dark brown, shield-shaped (Fig. 6B). Maxillae brown, longer than wide, with widened distal ends. Labium wider than long. Abdomen 5.69 long, 1.09 wide, slender, with dorsal silver guanine spots (Fig. 6A, B).

**Palp** (Fig. 6D−G). Paracymbium with strongly divided notch, translucent lobe occupying half of the paracymbium width and knob elbow-like and blunt. Cymbium distal half narrower than basal half in dorsal view. Tegulum oblate-spherical, sperm duct visible through cuticle. Conductor extending slightly beyond the distal end of the cymbium. Embolus encircling genital bulb clockwise for approximately three-quarters of a circle, distal half alongside and protected by conductor.

##### Notes.

*Tetragnatha hirashimai* Okuma, 1987 has impressive cheliceral fangs, especially in the female. This species had not been reported again in nearly four decades since it was described for the first time in 1987 based on specimens from New Guinea. The original description included numerous informative illustrations, such as habitus, abdomen, eye group, and ventral genital region of the female; chelicerae of both sexes; the paracymbium, and partial conductor and embolus of the male (fig. 26 in Okuma, 1987). However, the vulva was not illustrated, and the drawings of male palpal organ were not sufficiently detailed—particularly regarding key structures such as the embolus and conductor, where only distal portions were shown without showing a specified viewing angle.

Although we identified our specimens as *T. hirashimai* in this study, several differences between our specimens and Okuma’s should be recognized. First, the cheliceral fangs in females exhibit a subtle difference in shape. In the original drawings, the fangs are strongly bent, forming distinct dorsal projections at the midpoint (fig. 26F, G in Okuma, 1987), whereas in our specimen, the fangs lack dorsal projections and are smoothly curved at the corresponding position (Fig. 5D–F). Secondly, in dorsal view, the male cheliceral apophysis (a) is horizontally aligned with the Gu in our specimen (Fig. 6I), whereas in the original drawings, the ‘a’ (fig. 26A in Okuma, 1987) is positioned below the Gu, and the two structures are not horizontally aligned. However, it is crucial to note that the original description included only hand-drawn line illustrations provided by the author, which may differ to some extent from the actual structure or from photographs taken of the specimens.

We also noted that *T. mertoni* Strand, 1911, which was found in Indonesia, may represent a senior synonym of *T. hirashimai*. The two species appear highly similar based on the original illustrations (cf. fig. 45 in Strand, 1911 with fig. 26F, G in Okuma, 1987). Furthermore, given the known distribution of *T. hirashimai* in Papua New Guinea and its newly recorded presence in China in this study, it is plausible that this species also occurs in intermediate regions, such as Indonesia. However, since only a hand-drawn illustration of the chelicerae was provided in the original description of *T. mertoni*, and the types have not been examined, this issue remains unresolved and requires further investigation in future studies.

##### Distribution.

**Figure 7.**
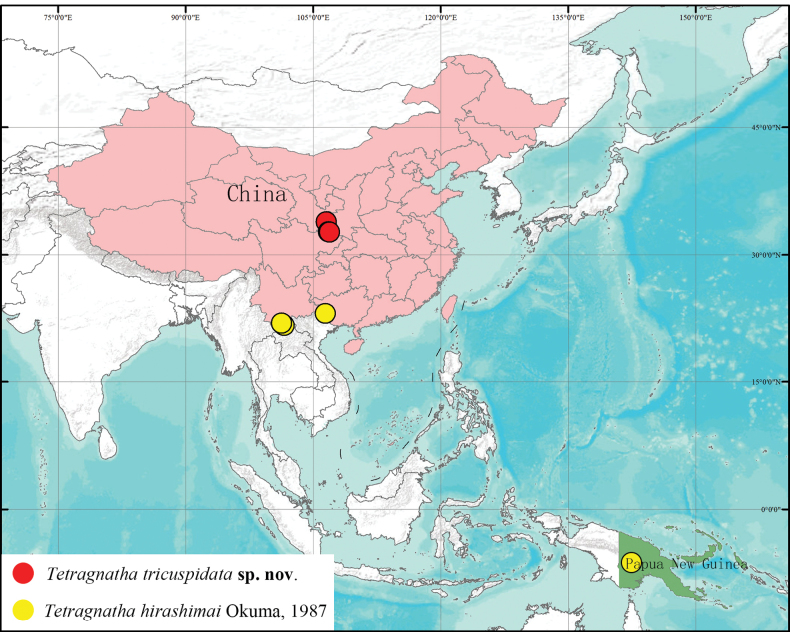
Distribution map of the *Tetragnatha* species in this study.

China (Guangxi Zhuang Autonomous Region, Yunnan, new record), Papua New Guinea (Fig. 7).

## Supplementary Material

XML Treatment for Tetragnatha

XML Treatment for Tetragnatha tricuspidata

XML Treatment for Tetragnatha hirashimai

## Additional information

### Use of AI

No use of AI was reported.

### Author ORCIDs

Changtao Liu https://orcid.org/0009-0000-0514-6402

Zongguang Huang https://orcid.org/0000-0003-1579-052X

Haiqiang Yin https://orcid.org/0000-0003-0276-2407

Xiang Xu https://orcid.org/0000-0001-9485-5373

### Data availability

All of the data that support the findings of this study are available in the main text.
